# Improving Dignity of Care in Community-Dwelling Elderly Patients with Cognitive Decline and Their Caregivers. The Role of Dignity Therapy

**DOI:** 10.3390/bs10120178

**Published:** 2020-11-24

**Authors:** Heifa Ounalli, David Mamo, Ines Testoni, Martino Belvederi Murri, Rosangela Caruso, Luigi Grassi

**Affiliations:** 1 Institute of Psychiatry, Department of Neurosciences and Rehabilitation (formerly Department Biomedical and Specialty Surgical Sciences), University of Ferrara, 44121 Ferrara, Italy; heifa.ounalli@unife.it (H.O.); david.mamo@um.edu.mt (D.M.); martino.belvederimurri@unife.it (M.B.M.); rosangela.caruso@unife.it (R.C.); 2 Department of Psychiatry, University of Malta, 2080 Msida, Malta; 3 Department of Philosophy, Sociology, Education and Applied Psychology (FISPPA), University of Padova, 35122 Padova, Italy; ines.testoni@unipd.it; 4 Emili Sagol Creative Arts Therapies Research Center, University of Haifa, Haifa 3498838, Israel

**Keywords:** dignity, elderly people, dignity therapy, cognitive impairment

## Abstract

Demographic changes have placed age-related mental health disorders at the forefront of public health challenges over the next three decades worldwide. Within the context of cognitive impairment and neurocognitive disorders among elderly people, the fragmentation of the self is associated with existential suffering, loss of meaning and dignity for the patient, as well as with a significant burden for the caregiver. Psychosocial interventions are part of a person-centered approach to cognitive impairment (including early stage dementia and dementia). Dignity therapy (DT) is a therapeutic intervention that has been shown to be effective in reducing existential distress, mood, and anxiety symptoms and improving dignity in persons with cancer and other terminal conditions in palliative care settings. The aims of this paper were: (i) To briefly summarize key issues and challenges related to care in gerontology considering specifically frail elderly/elderly with cognitive decline and their caregivers; and (ii) to provide a narrative review of the recent knowledge and evidence on DT in the elderly population with cognitive impairment. We searched the electronic data base (CINAHL, SCOPUS, PSycInfo, and PubMed studies) for studies regarding the application of DT in the elderly. Additionally, given the caregiver’s role as a custodian of diachronic unity of the cared-for and the need to help caregivers to cope with their own existential distress and anticipatory grief, we also propose a DT-dyadic approach addressing the needs of the family as a whole.

## 1. Introduction

Demographic changes have placed mental health, quality of life (QoL), and levels of functioning in the elderly at the forefront [1,2]. In Europe, where 30% of the population will be over 65 by 2050 [3], the impact of psychological disorders very common in old age, such as depression, anxiety, and somatoform disorders, are growing public health concerns [4,5,6]. These aspects are even more marked if we consider the problem of cognitive impairment and neurocognitive disorders (mild to moderate dementia and dementia), since these conditions markedly increase vulnerability and dependency and decrease the individual’s sense of self and dignity [7,8]. Therefore, intervention improving the areas of quality of life, [9] meaning in life [10] and dignity [11,12] should be considered extremely important when implementing good clinical practice services for the elderly population, particularly with mental disorders, including neurocognitive disorders.

Over the last decade, Dignity Therapy (DT) [13] has been shown to be a well-suited intervention for addressing existential issues at the end of life, such as dignity, meaning-making, continuity of self, preparation for death, and life completion tasks. While developed in the setting of palliative care with terminally ill patients, DT showed preliminary, yet interesting data among the elderly population, including those with cognitive decline. In effect, the cognitive, functional, and neuropsychiatric consequences of neurocognitive disorders can be equated to a life-threatening condition [14], as the individual’s sense of self, dignity, and personhood are at risk in the aforesaid conditions [15,16].

With respect to this, a person-centered approach that seeks to maintain dignity in older persons with cognitive decline [17,18] is not possible without including their informal caregivers, usually their close relatives. It therefore follows that targeting dignity and meaning intervention in the affected elder alone, outside of the context of the dyadic relationship with the informal caregiver, cannot be expected to be sufficient to improve mental health and quality of life in those affected by cognitive decline [19]. Therefore, the role of caregivers should be addressed in a more specific way when DT would be applied in a geriatric setting.

On these backgrounds, the aims of the paper were (i) to briefly summarize key issues and challenges related to care in gerontology considering specifically frail elderly and those with cognitive decline and their caregivers, analyzing the proposals for possible development of DT in the elderly setting, and (ii) to provide a comprehensive review of the recent knowledge and evidence on DT in the elderly population with cognitive impairment, with the intent of contributing to address a gap in therapeutic strategies targeting a key human interaction (dyad elderly/caregiver) and guarantee dignity-preserving care.

## 2. Methods

After accounting for DT and its efficacy in the area of palliative care and summarizing the relevant issues of dignity in the elderly with and without cognitive impairment, we proceeded to carry out the comprehensive analysis of studies applying DT to this population. The electronic databases CINAHL, SCOPUS, PSycInfo, and PubMed were extensively searched, using the terms ‘cognitive decline’ OR ‘ cognitive impairment’ OR ‘early dementia’ OR ‘dementia’ AND ‘dignity therapy’, and filtering for age ≥ 65 years.

### Data Extraction

Regarding the second aim of the paper, two investigators (HO, RC) independently examined all titles and abstracts and obtained full texts of potentially relevant papers using an ad hoc form. For all studies, we extracted information on study design, source of data, population characteristics, intervention details, and outcomes of interests.

## 3. Results

In the following sections, the literature evidence on implications of cognitive impairment on the elderly patients and their caregivers will be illustrated. Moreover, recent findings regarding the effectiveness of DT in the main filed of its application, namely end of life and palliative care, will be introduced as a premise to the following review on DT applied in gerontology. The findings of our comprehensive search on dignity-oriented interventions on this population (the second aim of the study) will be reported. A total of 296 articles were identified from electronic databases. Of these, 266 were excluded based on title and abstract screening, and the remaining 30 articles were retrieved for more detailed evaluation. A total of 5 individual studies eventually fulfilled the criteria for eligibility and were included in the review. Of these, one study was a mixed method randomized clinical trial, two were mixed method studies, and two were qualitative studies.

### 3.1. Dignity and Cognitive Impairment in the Elderly

As said, old age with the series of loss of function and decline in everyday activities is associated with problems that can impair the sense of dignity, as an inherent characteristic of human beings [11,12,20]. If institutionalized, elderly people may perceive a higher risk to lose their sense of dignity that has been shown to be threatened both in terms of intrapersonal dignity (who I am as myself) and relational dignity, which is socially constructed by the act of recognition and maintenance of social networks (who I am in relation with others) [21]. Being active to the very last, respecting one’s will and being allowed to die, not being in pain, and being amongst persons close to one were part of what the elderly felt to be factors increasing dignity [22]. Hall et al. [23], in a series of elderly patients specifically analyzed the sense of dignity as proposed by Chochinov et al. [24,25,26] in medicine (especially palliative care) and encompassing three main categories of dignity (Table 1). The authors confirmed that illness-related concerns, social aspects of the illness experience, and dignity conserving repertoire were part of the elderly experience.

#### 3.1.1. Implications of Cognitive Impairment on the Elderly Person

The age-related decline of cognitive ability is usually a slow process that has the potential to erode an individual’s identity and sense of “being in the world”, resulting in fragmentation and arguably a subsequent loss of the individual and the relational self in more advanced stages of cognitive impairment [27,28]. It is ascertained that a host of behavioral and psychological symptoms (e.g., sense of insecurity resulting in anxiety, frustration from inability to accomplish tasks, misidentification and misinterpretation of environmental cues, persons, and events, catastrophic reactions) in people with early stages of dementia become a source of profound distress for elderly patients with cognitive impairment, impacting and undermining their dignity. The sense of being in the world is disrupted every time episodic memories of new experiences are not created and the physical aspect of experiencing and autonomously interacting with the world itself declines, resulting in a dismantling and fragmentation of the self [29,30]. The dignifying aspects of care are in this respect represented by personalization, respect, attentiveness and encouragement, attention for physical care and bodily gestures, and fostering a sense of belonging [31]. In terms of dignity, the subjective perception of elderly people recently diagnosed with mild to moderate dementia has been shown to be characterized by some specific themes [32]. First, dissonance, represents the sense of progressive loss of competence becoming a threat to their identity and visibility, and thus a source of stigma. Although elderly persons tend to hide their diagnosis from others, including close family members, the competence decline in everyday tasks becomes increasingly apparent to the family and the wider social networks. A second theme, vulnerability, regards the feeling of being in a state of limbo as a significant source of distress determined by not knowing how much and when the functioning and performance status would further deteriorate. A third theme, loss of control and agency, is characterized by loneliness, increasing dependence, and a sense of becoming a burden, foreshadowed by diminished personal agency. Last, maintaining agency and self-worth are related to the process of accepting the diagnosis, adapting to changes using coping strategies, and accepting support from others. In this respect, maintaining dignity across the spectrum of both minor and major cognitive disorders, and supporting the narrative self-emerge as essential ingredients of successful aging in the course of cognitive decline [27,33].

#### 3.1.2. The Role of Caregivers and the Caregiving Process

The caregiver figure’s contribution to care has evolved significantly over the past decades, as a growing proportion of individuals assume caregiving duties more than once in a lifespan [34].

Specifically, in the elderly, functional and neuropsychiatric consequences of cognitive impairment create significant challenges for caregiving. A high burden of care falls in fact mainly on family members and, consequently, family caregiving is acknowledged as “the backbone” of elderly community care, especially for those with cognitive impairment [35]. Caregiver burden has been associated with psychological distress, interpersonal issues, and health disorders in caregivers of elderly patients with neurocognitive disorders, which in turn have been associated with sub-standard care and institutionalization. Caregivers of elderly people with mild cognitive impairment or dementia have been shown to be significantly more stressed than non-caregivers [36], with very high prevalence rates of depressive and anxiety symptoms (34% and 44%, respectively) in addition to a higher risk of other physical disorders [37]. Especially, but not only, behavioral and psychological symptoms secondary to dementia are correlated with caregiver burden and depression, with lower correlations for functional and cognitive impairment [38,39,40]. 

A further specific issue regards the phase following the loss of the patient, with a large literature available in geriatrics on anticipatory grief, bereavement, and complicated grief [41,42]. As far as neurocognitive disorders, a number of studies indicate the need to consider that grief and mourning for family members caring for their relative begin with the initial changes in the person’s cognitive abilities, and intensify with the worsening of his or her condition, and continue after death (Two-Track Model of Dementia Grief) [43]. Therefore, pursuing psychological health and well-being in informal caregivers, considering their effects on persons with neurocognitive disorders themselves and thus on health care costs, becomes a priority [44].

#### 3.1.3. Dyadic Approach to Care in the Elderly with Cognitive Decline

On these bases, recent research is reorienting the focus of intervention for the psychosocial implication of neurocognitive disorders by considering the dyad as a unit [45]. Various dyadic coping to stress models have emerged based on the assumption that stress and coping are reciprocal, where cognitive and emotional appraisal of a stressor and coping responses are embedded at an interpersonal level. In effect, positive dyadic coping is significantly associated with relationship satisfaction, regardless of partner’s socio-demographic and cultural characteristics or relationship duration [46]. In line with this, authors of a recent meta-ethnography on dyadic construction of dementia further ascertained that dyad-set goals leading to a “co-responsibility and ownership” of the dementia experience promoted positive interaction, [47] while “hostile/ambivalent coping”, “protective buffering”, and “overprotection” were retained negative dyadic coping styles [48]. For these reasons, a dyadic approach in early-stage, community-based dementia care (e.g., dual support groups, dyadic counselling, cognitive stimulation, skill training, and multi-dyad memory notebooks), has shown to be well accepted and to improve communication, interpersonal relationships, and quality of life among both caregivers and care-recipients [49]. Other reviews investigating the effectiveness of dyadic psychosocial programs for community dwelling elderly with neurocognitive disorders and their family caregivers further confirm the positive outcomes of dyadic approach (e.g., improvement of behavioral symptoms, mood disturbances, and activities of daily living in the patient, and reduction of distress and burden in the caregiver) [50]. 

### 3.2. Psychosocial Intervention in a Dyadic Approach: The Role of Dignity Therapy

Over the last decades, a number of psychosocial interventions have been developed, within a person-centered care framework (PCC), to help elderly with neurocognitive impairments in long-term facilities to preserve their sense of identity and selfhood [51,52,53]. A series of reviews and metanalysis have examined the role of reminiscence therapy, life review, or other forms of intervention based on a chronological review and evaluation of life experiences, with data generally underlining the efficacy of these intervention, [54] while some indicate less consistent results [55]. 

With respect to this, Dignity Therapy (DT) is a form of intervention that, within a PCC, can have a specific role in the elderly with neurocognitive disorders. Unlike other psychosocial approaches in the elderly (e.g., life review, reminiscence therapy), DT does not have the aim to produce a standard chronological biography neither to improve the memory, but essentially to focus on significant events and relationships in life which give the person a sense of meaning and pride, to recall the most important roles played in one’s own life or important accomplishments and lessons learned about life to be pass along to others. This sense of generativity, as a written and permanent record of the patient’s thoughts and words, is also part of the dignity model and DT, and is instrumental to the improvement of the communication between the patient and the family, as described below.

#### 3.2.1. The Structure of Dignity Therapy

As said, DT has been initially developed in palliative and cancer care as one of the forms of psychotherapy at the end of life [56]. It is a short manualized intervention based on Chochinov’s conceptualization of dignity, and it is an alternative approach to bypass the limitations of other interventions (e.g., psychodynamic, interpersonal psychotherapy) due to the time and functional limitations inherent in end-of-life care. [13]. Therefore, DT is primarily guided by specific subthemes of the dignity model, including continuity of self, maintenance of pride and hope, role preservation, generativity, and aftermath existential concerns. Through DT, a dying person is provided the opportunity to explore aspects of life that are perceived as most meaningful, identify aspects of self and personal history for which they wish to be remembered, take the opportunity to express what they feel needs to be said before dying, and create a written lasting legacy to pass on to their identified loved ones. In broaching the areas elicited by the DT framework, the patient is guided by the therapist through a semi-structured interview (Table 2), which is tape-recorded, transcribed verbatim, and shaped into a narrative through a preliminary editing process. During the following session, the participant is invited to further edit the written document as desired. The final generativity document is thus given back to the participant to share it with family members or loved ones.

Since the first original Randomized Clinical Trials (RCTs) conducted in palliative care settings and showing the efficacy of DT in reducing sadness and depression and increasing dignity and will to live in terminally ill patients [57,58], DT has been the focus of a growing body of research, with both quantitative as well as qualitative data now available in palliative care settings.

Reviews of the many studies carried out in the last years in at least eleven countries confirm that DT is feasible and overall accepted in various clinical and cultural realities, and it is effective in promoting patient/family sense of dignity, meaning, and quality of life, while reducing psychological and dignity related distress [59,60,61,62,63]. A recent meta-analysis examining ten RCT and confronting DT applied in cancer palliative care to standard care demonstrated that DT significantly decreased anxiety, depression, and dignity related distress in advanced cancer patients [64]. A different meta-analysis opposing DT to control conditions further evidenced that DT promoted dignity, existential, and social support domains [60]. DT has also been applied in patients with other diseases rather than cancer, including motor neuron disease [65] and advanced chronic obstructive pulmonary disease [66].

#### 3.2.2. Dignity Therapy as Applied in the Elderly Population and Early Dementia Patients

While growing old should not be equated with a terminal illness, the gradual transition for mid-life to late-life involves normative psychological transition issues associated with an implicit life review and negotiation of losses and missed opportunities. The data about the application of DT in palliative settings considering the available solid empirical evidence in that field can be therefore helpful in gerontology. In fact, DT background represents a solid basis of the rationale of broadening the use of this intervention in affiliated fields sharing similar dignity related, existential, and psychosocial implications among frail elderly with cognitive impairment. Palliation is a concept that should be not only intended as an intervention for terminally ill patients, but as a more general construct related to support and palliate the several dimensions of suffering in different clinical areas [67], including geriatrics, psychiatry, and others [68,69].

Among the elderly population, the dignity model lends itself well to assisting elderly individuals to understand their self through clarification of their life’s values and meaning as death approaches [70] since a review of one’s life experience is universal in older persons.

Recent research is investigating the DT applicability perspectives in gerontology, especially in frail elderly and cognitive impaired individuals. Although available data are still limited, preliminary promising findings emerged in the elderly population, with studies carried out in patients with and without cognitive decline reporting a positive effect on dignity, as summarized in Table 3.

In one of the first studies, Chochinov et al. [71] applied DT in 23 care home residents including 12 cognitively intact residents (>21 on the Mini Mental Status Examination) and 11 cognitively impaired residents who were represented by way of family member proxies. Interestingly, the narratives of both cognitively intact participants and the family proxies of residents with cognitive impairment shared several common themes, namely “imparted life lessons”, “importance of relationships”, “source of pride or accomplishment”, and “delights or joys”. The outcome was assessed using a post-intervention questionnaire for both residents, family members, family proxies, and health care providers. The majority of the participants found the therapy helpful or thought that it was of comfort for their loved one. Health care providers (HCP) not only perceived the therapy as helpful for providing better care to residents, but also reported that it deepened the way they knew the resident as a result of new insights on the individual that resulted from the DT. This was confirmed by the family proxies involved in DT sessions as narrators of the meaningful life story and the valuable legacy of their loved ones as further outlined by “personal characteristics” and “important roles” themes. Nonetheless, unlike HCP, family members could not expressly evaluate DT outcomes in terms of enhanced sense of meaning, purpose, and dignity, or alleviated suffering among participants. They rather described DT as a valuable moment for enhanced communication, gaining of insight and valorization of their loved one’s life, as well as an ongoing source of comfort to them over time.

A randomized controlled study carried out by Hall et al. [72,77] used a sample of 60 frail cognitively intact older residents in a nursing home in the UK. DT did not differ significantly from standard psychological and spiritual care in outcome measures related to dignity perceived distress, depression, hopefulness, or quality of life, although both groups showed a significant decrease in Patient Dignity Inventory (PDI) scores compared to the baseline, and both therapeutic approaches were perceived to be helpful by the family. Moreover, DT bolstered participants’ sense of meaning, purpose, and will to live, and alleviated their suffering with effect sizes ranging from small to medium at 8 weeks follow-up. The authors outlined that valorizing the achievements of residents and treasuring these in their generativity documents seemed to enhance sense of pride and hopefulness. The care tenor was evident as participants expressed enjoyment for the visits of therapists and researchers [73]. Moreover, in line with the findings from Chochinov et al.’s study [71] some DT participants further highlighted the benefits of generativity documents as a valuable moment of positive sharing and enhanced communication both with families and health professionals [73]. This study, however, was limited by low levels of baseline perceived dignity distress, high levels of hopefulness in both groups, as well as the dropout resulting in loss of study power. Feasibility issues were also raised by the authors, as DT among elderly residents might result as more time consuming compared to other clinical settings.

From a qualitative point of view, Goddard et al. [74], among a sample of 14 generativity documents’ recipients (mainly family members) of 27 elderly people in home care, showed that DT helped residents reappraise aspects of their lives positively, while at the same time enjoying the opportunity to reminisce. Additionally, family who received the generativity document appreciated the opportunity of deepening their knowledge of their loved one, and cherishing and passing along the permanent tangible legacy. Bereaved family members also acknowledged that documents provided them comfort during their grief. On these bases, the authors concluded that DT may be a useful intervention for enhancing the end-of-life experience for both residents and their families. Of note, however, few family members and/or residents expressed concern over document content, especially in case of eventual negative references, omissions, inaccuracies, or ongoing family issues, as also outlined by some residents from Hall et al. RCT [73]. It is, however, relevant to outline that the editing DT session is fundamentally conceived as an opportunity to address freely such issues by editing, adding, and deleting content as desired.

Johnston et al. [75] assessed the feasibility of DT in early stage dementia in a small mixed-methods study including seven community-dwelling individuals with early stage dementia, seven stakeholder participants, and six focus group members. They noted that the intervention and the outcome measures used, including the PDI, were appropriate, although more time was required to complete the therapy and edit the generativity document compared to other palliative care settings. Importantly, at this stage, the cognitive decline of participants did not seem to interfere with the intent of DT to capture relevant and meaningful aspects of self and of one’s life story. The interviews were conducted with both patient and family members present, and the latter often helped to prompt and confirm events. The authors provided evidence that the DT is feasible and acceptable to elderly people with early stage dementia. In qualitative in-depth analyses of the themes emerged during DT, three main themes emerged in the documents: “A life in context”, “a key to connect”, and “personal legacy”. Regarding outcome measures, although the sample size was limited, two, three and further two participants reported higher Herth Hope Index (HHI) and quality of life scores and lower PDI scores (i.e., reduced dignity related distress), respectively among a total of 4 participants fulfilling pre and post DT follow up assessments. In a depth analysis of DT narratives, the origins of the individuals’ values, essence and affirmation of the self, forgiveness and resolution, existentialism, and meaning of life also emerged as further sub-themes [76]. In a further recent qualitative study carried out in institutionalized elderly suffering from multiple comorbidities, a modified version of DT was applied by also using an album of family photographs. It has been shown that the use of a photo album facilitated the recollection of facts and emotions, and reflection on the values that shaped the narrated content and that constituted the generativity document [78]. The main results of the DT studies available in the elderly settings are reported in Table 2.

#### 3.2.3. The Need to Involvement of the Family in DT in Geriatrics

Although studies of DT in the elderly sometimes involved family members, no specific data are available on the dyads as a constant approach for DT in this field. However, the benefit of involving dyads and family members emerged in studies carried out, as already described, in the context of palliative care. In this area, reviews of the research available indicate that the involvement of the family varied from a supportive/comforting presence during sessions to a more proactive contribution in the narrative and/or editing process where requested [62]. What emerges by the review is that most of family members treasure DT generativity documents as an ongoing source of comfort accompanying them through the (anticipatory) grief process and a helpful intervention to tackle stress and nurture hope for the future. Furthermore, most of the family acknowledge the benefits of DT on their loved ones and would recommend it as a key moment in care. Recent trials are in progress to examine this area more specifically [79], in order to address the problem of creating a sense of coherence between the patient and the family when working on dignity and the issues related to the meaning of their own life [80].

A further interesting perspective/lecture in favor of dyadic approach to dignity in elderly could also be deduced from the studies actively involving family members in the DT process. For instance, family proxies participating on behalf of cognitively impaired residents valorized their “personal characteristics” and “important roles” [71]. These themes were much less represented in the narratives of cognitive intact participants while narrating themselves. Such contributions could be considered as a memory-prone helping to collect further meaningful life moments and aspects of self-regarding the resident. Furthermore, it could be a valuable reciprocated legacy bolstering patient’s continuity of self, role preservation, self-identity, and generativity as acknowledged by the family member/caregiver.

With respect to this and what is said above regarding the role of the family towards elderly people with early dementia, it is important to consider a DT in a dyadic approach. In effect, DT working in a dyadic approach could favor the quality of relationship between the patient and their caregiver, with possible improvement in both patient’s outcomes, such as cognitive and functional decline, institutionalization and quality of life, as well as the caregiver’s well-being [81]. Support of the caregiver–cared for dyad within DT emerges as a cornerstone in the disease coping process where mutuality (i.e., positive engaging and emotional support) and connectedness assume a crucial importance in determining psychological well-being of both patient and caregiver [11,82]. Additionally, as already said, the time following the loss of their loved one, after his or her death, also has significant meanings in terms of the bereavement process [83]. Thus, it would be important to examine the effects of a dyadic approach of DT with respect to this area. The identification of these themes in a personalized account—the generativity document—might provide a prompt to connect with the individual even at a later stage of the illness, and may help face behavioral manifestations that emerge in dementia—that it is, unmasking the “person” behind the behavior may improve communication and help the caregiver better understand and respond appropriately and effectively.

## 4. Discussion

Age-related cognitive impairment and deterioration are a significant common problem and a global public health issue, with the prevalence of neurocognitive disorders expected to rise exponentially due the aging populations. While not a ‘terminal illness’ per se, a palliative approach has long been advocated since cognitive degeneration and the progressive physical, functional, and neuropsychiatric difficulties threaten the tenets of personhood and dignity such that it is not unreasonable to consider it a ‘life threatening’ condition [11,16]. Psychological interventions with the aim of maintaining a sense of meaningful self, notwithstanding its apparent gradual disintegration, are clearly an ethical and clinical imperative [31].

On the other hand, as said, the unravelling of the self cannot be considered in isolation: Not only are informal caregivers often the only potential means of ensuring the preservation of dignity and personhood, but they also experience a parallel existential and psychosocial suffering that is associated with significant physical and mental morbidity. Since the construction of meaning, experiences, and identities is an interpersonal process, approaching the caregiver and cared for person as a dyad, where the continuity of the interwoven selves lies at the core of care practices, is imperative [46].

Within this perspective, global and national agendas regarding neurocognitive disorders, and especially dementia, have stressed the importance of the preservation and promotion of patients’ dignity, and the importance of taking into account the family emotional needs, DT can be considered a valid clinical approach for study in cognitive disorders in later life. DT, as a brief narrative and meaning-based intervention, has been applied within end-stage diseases and chronic life-threatening conditions, with a growing body of research thus confirming its solid empirical base across a variety of cultural realities. The literature concerning its use and efficacy in older persons and/or patients with cognitive impairment, though limited, has suggested it is feasible and well-received by both the patients and their formal and informal caregivers. Likewise, with elderly participants who reported subjective benefits after DT, the majority of family members acknowledged the potential benefits of this intervention on their loved ones and underlined the role of the generativity document as an ongoing source of comfort.

This study has some limitations to be considered. In first place, this is not a systematic review, and consequently it does not endeavor to follow the same methodological rigor and systematicity. Secondarily, the number of studies retrieved and the sample size included are limited. As a third limitation, the heterogeneity of study designs and populations must be mentioned. Most of the trials are pilot studies investigating the applicability and acceptability of DT in gerontology with a focus on qualitative—though nonetheless valuable—data. Nonetheless, we believe that this study could represent an important contribution in valorizing dignity-oriented interventions in the care of frail elderly patients.

## 5. Conclusions

Further research is still needed to appraise the DT outcomes in terms of efficacy and benefits for both the frail elderly, especially if with neurocognitive disorders, and the caregivers [42,43,84]. It is clear, as Chochinov [13] indicated, that the introduction of DT to the geriatric population is fraught with challenges since elderly individuals may not typically perceive themselves to be dying, therefore leaving it uncertain whether the intervention resonates as meaningful or fitting, and cognitive impairment should be evaluated in terms of having the chance to respond accurately and reflectively on a broad range of important and meaningful issues. For these reasons, elderly people with early dementia can be part of DT intervention, as the last chance for them to work in the reminiscence of significant and meaningful events of their past to leave to their loved ones as a lesson, hope for the future and legacy. As Johnston et al. [70] suggest, DT uses a skilled, trained dignity therapist to guide the person with dementia or early dementia through an interview that encourages discussion about the people and life events that have been important to them. In this sense, we propose that dignity-in-care and DT offer a unique opportunity as a psychotherapeutic intervention in the geriatric setting, including the care of people with early stage dementia, and suggest the adaptation of DT so that it embraces the dyadic aspect of the caregiving experience with the aim of promoting family connectedness and cohesion. The key-caregiver will in fact be asked to reflect upon meaningful life experiences and valuable lessons transmitted by the patients, and share messages of love and pride regarding the latter. The underpinning intent is to further bolster the patient’s continuity of self, role preservation, self-identity, and generativity as acknowledged by the caregiver. Moreover, DT may furnish a structured setting for caregivers to reciprocate words of affection, love, and wisdom so far received from their loved relative.

It would be important to train health care professionals and the staff working with elderly people in DT, as proposed for palliative care settings [85], to increase the likelihood to have a dignity-in-care framework within the facilities for the elderly and the application of short but enriching interventions, such as DT, in these facilities

Such an approach may contribute to address a gap in our therapeutic strategies targeting a key human interaction that holds the promise of preservation of personhood and dignity in elderly people with cognitive negative line as well as the physical and psychological well-being of the caregiver across the span of caregiving and beyond.

## Figures and Tables

**Table 1:** Framework of dignity components.

Illness-related concerns: Concerns related to the level of independence (cognitive acuity; functional capacity) and symptoms distress (physical distress; psychological distress; medical uncertainty; death anxiety) Dignity-conserving perspectives (self-continuity, role preservation; generativity/legacy; maintenance of pride; hopefulness; autonomy/control; acceptance; resilience/fighting spirit) and dignity-conserving practices (living in the moment, maintaining normalcy; seeking spiritual comfort) Social aspects of dignity: Privacy boundaries, social support, care tenor, burden to others, aftermath concerns.

**Table 2:** Framework of questions used in Dignity Therapy (DT) modified for the elderly setting.

Tell me a little about your life history; particularly the parts that you either remember most, or think are the most important? (Another way of putting this, which may elicit answers from some, is to ask, when did you feel most alive?) Are there particular things that you would want your family to know about you, and are there particular things you would want them to remember? What are the most important roles you have played in your life (family roles, vocational roles, community service roles, etc.)? Why were they so important to you, and what do you think you accomplished within those roles? What are your most important accomplishments, and what do you feel most proud of? Are there particular things that you feel still need to be said to your loved ones, or things that you would want to take the time to say once again? What are your hopes and dreams for your loved ones? What have you learned about life that you would want to pass along to others? What advice or words of guidance would you wish to pass along to your (son, daughter, husband, wife, parents, other(s))? Are there words or perhaps even instructions you would like to offer your family, in order to provide them with comfort or solace? In creating this permanent record, are there other things that you would like included?

**Table 3:** Available studies applying DT in the elderly population.

Authors	Type of study	Sample	Results
Chochinov et al. (2012) [71]	A mixed method clinical trial	12 cognitively intact and 11 cognitively impaired subjects	Patients: Imparted life lessons (5/12); importance of relationships (8/12); sources of pride or accomplishment (8/12); delights or joys (8/12); imparted life lessons (6/11); importance of relationships (9/11); sources of pride or accomplishment (8/11); delights or joys (10/11).
Hall et al. (2012; 2013) [72,73]	A mixed method randomized controlled open-label trial	31 subjects in DT and 29 standard care (psychological and spiritual care)	Significant overall reduction in dignity-related distress across both groups. At T2, small effects in favor of the intervention group on depression and quality of life (EQ-5D only). DT made life for participants more meaningful at 2-week follow-up and helped the families at both follow-ups. Qualitative analysis. Among DT participants, 3 main themes: Views on the generativity document; generativity; and reminiscence.
Goddard et al. (2013) [74]	Qualitative (Semi-structured qualitative interviews)	14 Family members	Patients: Good Interaction with the therapist; positive reappraisal: reflection on their life and their achievements. Reminiscence: Opportunity to talk about their past. Family: Acquiring new knowledge about their loved one; useful in bereavement.
Johnston et al. (2016) [75]	Feasibility mixed method study	27 (7 with ESD, 7 family members, 7 stakeholder participants + 6 in focus groups)	Positive changes in Herth Hope Index (HHI); Patient Dignity Inventory (PDI); and Perceived Quality of Life, and Satisfaction with Quality Life Ratings.
Johnston et al. (2017) [76]	Qualitative	7 patients with dementia	Seven themes during DT: Origin of values; essence and affirmation of self; forgiveness and resolution; existentialism/meaning of life.

DT: Dignity Therapy; ESD: Early stage dementia.
